# Disturbance‐mediated invasions are dependent on community resource abundance

**DOI:** 10.1002/ecy.3728

**Published:** 2022-06-01

**Authors:** Luke Lear, Daniel Padfield, Hidetoshi Inamine, Katriona Shea, Angus Buckling

**Affiliations:** ^1^ College of Life and Environmental Science University of Exeter Penryn UK; ^2^ Department of Biology and Center for Infectious Disease Dynamics, 208 Mueller Laboratory The Pennsylvania State University University Park Pennsylvania USA

**Keywords:** biodiversity, disturbance frequency, invader life history, invasion, invasion success, resource abundance

## Abstract

Disturbances can facilitate biological invasions, with the associated increase in resource availability being a proposed cause. Here, we experimentally tested the interactive effects of disturbance regime (different frequencies of biomass removal at equal intensities) and resource abundance on invasion success using a factorial design containing five disturbance frequencies and three resource levels. We invaded populations of the bacterium *Pseudomonas fluorescens* with two ecologically different invader morphotypes: a fast‐growing “colonizer” type and a slower growing “competitor” type. As resident populations were altered by the treatments, we additionally tested their effect on invader success. Disturbance frequency and resource abundance interacted to affect the success of both invaders, but this interaction differed between the invader types. The success of the colonizer type was positively affected by disturbance under high resources but negatively under low. However, disturbance negatively affected the success of the competitor type under high resource abundance but not under low or medium. Resident population changes did not alter invader success beyond direct treatment effects. We therefore demonstrate that the same disturbance regime can either be beneficial or detrimental for an invader depending on both community resource abundance and its life history. These results may help to explain some of the inconsistencies found in the disturbance‐invasion literature.

## INTRODUCTION

Biological invasions are a global issue with potentially severe consequences for native communities (Davis et al., 2000; Fausch et al., 2001; Lake & Leishman, 2004; O'Dowd et al., 2003). Successful invader colonizations can reduce biodiversity, alter community dynamics, and cause large financial costs (Altman & Whitlatch, 2007; Didham et al., 2005; Fausch et al., 2001; Leishman et al., 2007; Levine et al., 2003; Shea & Chesson, 2002; Sher & Hyatt, 1999; Vitousek et al., 1997). Disturbances—events that, through destroying biomass, change the availability of resources and habitats—often promote invader success (Altman & Whitlatch, 2007; Lear et al., 2020; Roxburgh et al., 2004; Shumway & Bertness, 1994). Disturbances can vary in frequency (how often they occur in a given time period), extent (e.g., small, such as leaves falling, or large, such as wildfires), timing, intensity (proportion of biomass removed), and duration (e.g., long term [press] or brief [pulse]) (Miller et al., 2021), and facilitate invasions in a number of ways, for example by increasing resource availability, which in turn reduces invader–resident competition (Baldwin & Mitchell, 2000; Davis et al., 2000; Hobbs & Huenneke, 1992; Lake, 2000; Lear et al., 2020; Tilman, 2004). Disturbances may also alter any priority effects, impact community succession and cause resident maladaptation (Altman & Whitlatch, 2007; Davis et al., 2000; Fargione et al., 2003; Fukami, 2015; Stachowicz et al., 2002).

Despite a large body of work showing that disturbance increases invader success (Altman & Whitlatch, 2007; Lake & Leishman, 2004; Lear et al., 2020; Lembrechts et al., 2016; Roxburgh et al., 2004), some studies show no or even a negative effect (Fausch et al., 2001; Narimanov et al., 2020). This may be due to disturbance interacting or covarying with other key environmental variables that affect success. Resource abundance is likely to be particularly important in this context (Davis et al., 2000; Lear et al., 2020). Where resources are abundant but not easily accessible, disturbance is likely to play an important role in promoting invader establishment. This is because disturbance will lead to an increased availability of resources that would otherwise be stored as biomass and depleted by consumption (Davis et al., 2000). In communities with low resources, the amount of resource released by disturbance will necessarily be low (Davis et al., 2000). The relative change in resource abundance may be equal between resource abundant and resource poor environments following a disturbance, but the absolute amount released by disturbance will be higher in a resource abundant environment.

The effects of disturbance and resource abundance on invasion success are likely to depend on the invader's life history traits (Roxburgh et al., 2004). Specifically, successful invaders are often fast‐growing “colonizer” species (van Kleunen et al., 2010) that can quickly convert available resources into biomass (Mächler & Altermatt, 2012), and so are expected to excel in high disturbance and resource abundant conditions. However, whether slower‐growing “competitor” species invade more successfully at low disturbance and low resource abundance remains unclear.

Disturbance and resource abundance may have additional indirect effects on invasion by altering the composition of the resident community. On the one hand, disturbance frequency and resource abundance can help increase community productivity and biodiversity (Agard et al., 1996; Kassen et al., 2004; Worm et al., 2002), which in turn may make the community more resistant to invasion (Brockhurst et al., 2006; Hodgson et al., 2002; Levine & D'Antonio, 1999; Tilman, 2004): productive and diverse communities are more likely to contain dominant species (e.g., species that have a disproportionally large influence on invasion resistance) and have priority effects (i.e., situations where the first species to occupy a niche has a fitness advantage over species arriving subsequently) (Fargione et al., 2003; Fukami, 2015; Hodgson et al., 2002). These factors increase invasion resistance mainly by reducing invader access to resources (Emery & Gross, 2007; Fukami, 2015; Hodgson et al., 2002; Naeem et al., 2000; Seabloom et al., 2003; Tilman, 2004). On the other hand, there is growing evidence that greater levels of diversity may facilitate invasions through increased niche dimensionality (Green et al., 2011; Ricciardi, 2001; Simberloff & Von Holle, 1999), which increases the chance of an invader occupying a niche and leads to a negative relationship between diversity and invasion resistance (Fridley et al., 2007). Disturbance may weaken or eliminate these effects of residents (dominant species, priority effects, and diversity‐derived niche dimensionality) by decreasing resident population sizes, opening up niches, and causing resource influxes.

Here, we experimentally investigate the independent and interactive effects of resource abundance and disturbance on invader success. We do this by invading wildtype populations of the bacterium *Pseudomonas fluorescens* SBW25 with genetically marked *P. fluorescens* SBW25 genotypes (Hodgson et al., 2002; Lear et al., 2020; Zhang & Buckling, 2016) at different disturbance frequencies and resource abundances in a fully factorial design. Invading with the same species as the resident population allows us to assume any differences in invader success are solely due to treatment effects, and not differences in resident and invader fitness. The rapid evolutionary diversification of *P. fluorescens* populations into three distinct niche specialists (Gómez & Buckling, 2013; Rainey & Travisano, 1998) allowed us to determine any additional effects of evolved biodiversity and resident density—caused by disturbance and resource variation—on invasion success (Hall et al., 2012; Kassen et al., 2000; Koza et al., 2011; Rainey & Travisano, 1998; Spiers et al., 2002). Although this is a highly simplified “community” with relatively little phenotypic variation, diversity–disturbance relationships in this system (Buckling et al., 2000) correspond with those in more complex and natural microbial systems (Galand et al., 2016; Zhang et al., 2018); it therefore offers a useful insight into what may happen in more complex communities. We invaded resident populations with two distinct genotypes: a fast growing, colonizer morphotype and a slower growing competitor morphotype (Hall et al., 2012). The difference in growth rates between these genotypes is due to the competitor type investing in biofilm formation, which allows it to colonize the oxygen‐rich niche near the air–liquid interface; this carries the cost of a significantly reduced growth rate compared to the colonizer type (Koza et al., 2011; Spiers et al., 2002). This allowed us to determine whether the effects of disturbance and resource abundance on invasion success was contingent on the invader's life history.

## METHODS

### Resident populations

**FIGURE 1:**
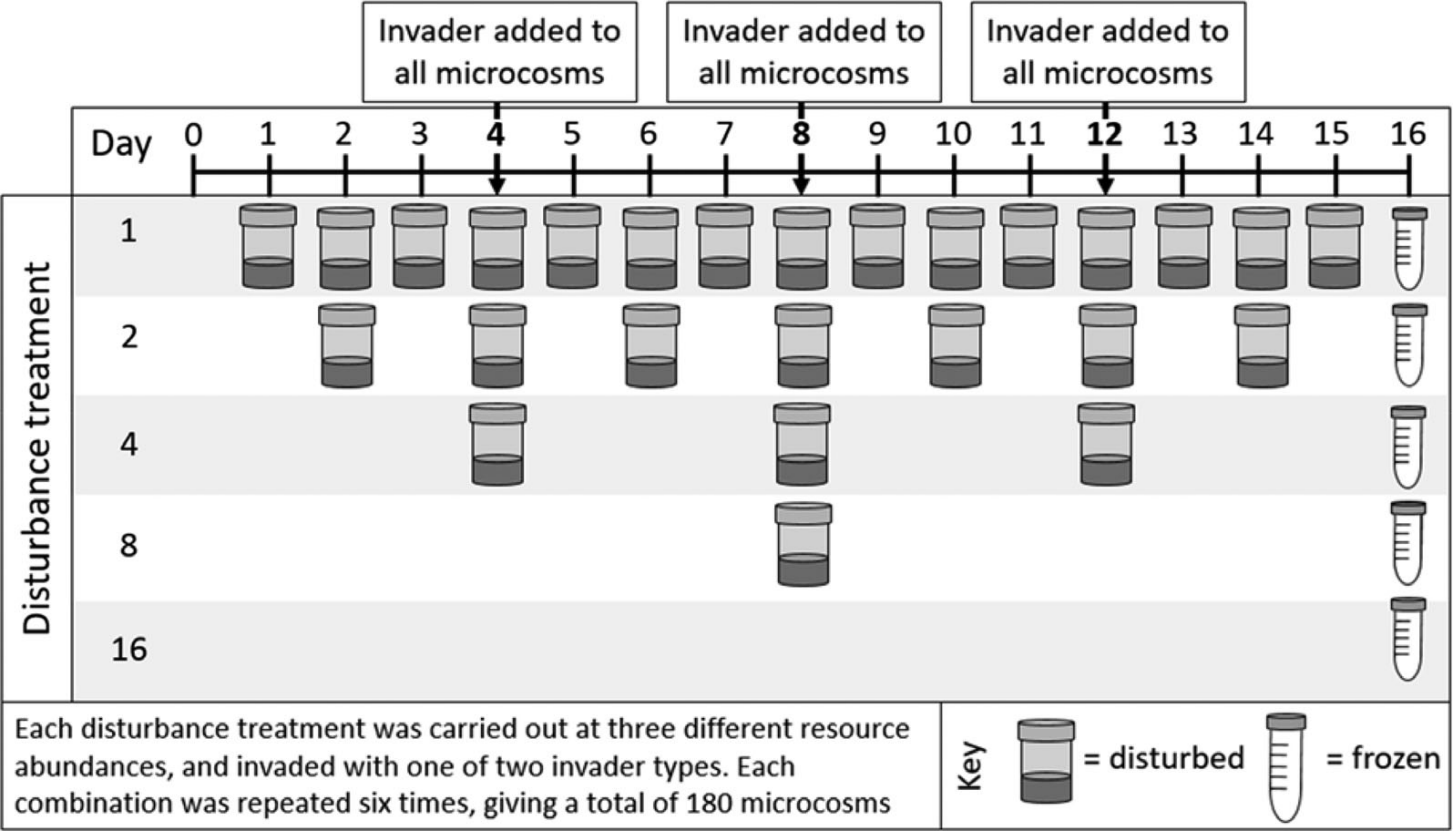
Schematic of the experimental design. Microcosms of either 100%, 10%, or 1% resource concentration were disturbed every 1, 2, 4, 8, or 16 days (denoted by an icon of a microcosm) to test for the effects of both disturbance frequency and resource abundance on invader success. Disturbances involved 1% transfer of homogenized broth into fresh media. All microcosms were invaded every 4 days (immediately post‐disturbance) with either a smooth (SM) or wrinkly spreader (WS) invader. Six replicates per treatment were used.

Ancestral *Pseudomonas fluorescens* SBW25 was grown overnight to carrying capacity in shaken glass vials (microcosms) containing 6 ml of King's medium B (KB) at 28°C. 60 μl of this culture was then transferred into static microcosms containing KB of varying concentrations (100% KB, 10% or 1%) to create different resource abundances; KB was diluted with M9 salt solution (3 g KH_2_PO_4_, 6 g Na_2_HPO_4_, and 5 g NaCl L^−1^). Five disturbance treatments were used, with microcosms disturbed every 1, 2, 4, 8, or 16 days (Figure 1) by transferring 1% of homogenized broth into fresh media (99% mortality) for a total of 16 days. Disturbing in this way results in pulse‐type disturbances with equal disturbance intensity across all treatments, as 1% of all populations will survive regardless of their density. Invaders were inoculated at days 4, 8 and 12 (Figure 1 and see *Invasions*). In between transfers and invasions, all microcosms were kept static at 28°C with loose lids to allow oxygen transfer. We used 12 replicates of each resource abundance (3) and disturbance frequency (5) combination, for a total of 180 microcosms. Additional microcosms (*n* = 3 per resource abundance × disturbance combination) were set up to quantify resident density on day 4: the first invasion time point. This was necessary as the sampling microcosms required homogenization of treatments that would otherwise not be disturbed.

### Invasions

*Pseudomonas fluorescens* with a *lacZ* marker was used as the invader; the *lacZ* marker makes it visually distinguishable from the wildtype on agar containing X‐gal (5‐bromo‐4‐chloro‐3‐indolyl‐β‐d‐galactopyranoside) due to a blue color change (Zhang & Rainey, 2007). Although originally reported as a neutral marker (Zhang & Rainey, 2007), the *lacZ* insert has previously been found to offer a fitness advantage to invaders (Lear et al., 2020). The strain was left to diversify for 5 days in static KB before being plated and a single smooth (SM) and wrinkly spreader (WS) colony was selected, grown and stored in 25% glycerol solution at −80°C. SM morphotypes (our “colonizer” invader) inhabit the broth where they grow rapidly, whereas WS (our “competitor” invader) form biofilms at the air–broth interface: biofilm formation increases competitive ability for oxygen but at a cost to growth rate (Hall et al., 2012). Before each invasion, these freezer stocks were used to grow each morphotype overnight in shaken KB as described in *Resident populations*; these cultures were diluted to 1% with M9 salt buffer before use. All microcosms were invaded every 4 days with 60 μl of either SM or WS invader (total colony forming units [CFU] added over the three events: SM = 8.1 × 10^6^; WS = 6.6 × 10^6^). If a microcosm had been disturbed, invasion would occur post‐disturbance. This resulted in six replicates for each disturbance by resource abundance combination per invader morphotype.

Experiments finished on day 16, when all microcosms were homogenized and a 900 μl sample was frozen in 25% glycerol at −80°C. After plating on KB agar containing 100 μg/L of X‐gal, wildtype, and invader SM, WS and fuzzy spreader (FS; a rarer bottom‐dwelling morph; Rainey & Travisano, 1998) colonies were counted.

### Statistical analyses

All counts were first standardized to colony‐forming‐units (cfu) per ml. Invasion success (relative invader fitness) was calculated as proportional change, *v*, of the proportion of invader to resident, calculated as *v = x*_2_.(1 − *x*_*1*_)/*x*_1_.(1 − *x*_*2*_), where *x*_1_ is the initial invader proportion and *x*_2_ the final (Ross‐Gillespie et al., 2007). Initial invader proportion (*x*_1_) was calculated as the average frequency of the introduced invader:

(1) $$x_{1}=E\left[\frac{I_{t}}{I_{t}+R_{t}}\right]=\frac{1}{3}\sum_{t=\left\{4,8,12\right\}}\frac{I_{t}}{I_{t}+R_{t}}$$

where *I*_*t*_ is the density of the invader introduced on day *t* and *R*_*t*_ is the density of the residents getting invaded on day *t*. We could not measure resident density on days 8 and 12, because it would require destructive sampling of undisturbed treatments. We therefore used the resident density on day 4 and assumed that *R*_4_, *R*_8_, and *R*_12_ were equal for 1‐, 2‐, and 4‐day disturbance treatments.

We sampled *R*_4_ for 1‐, 2‐, and 4‐day disturbance treatments during their transfers, but we could not sample *R*_4_ for 8‐ and 16‐day disturbance treatments, as it is a destructive process. The disturbance history up to day 4 for 8‐ and 16‐day treatments is identical to that for 4‐day treatment. We therefore assumed the resident community dynamics are the same for these three treatments, and used *R*_4_ for 4‐day treatment (before the disturbance) to calculate *R*_4_ for 8‐ and 16‐day treatments:

(2) $$R_{4,8-\text{days}}=R_{4,16-\text{days}}=\frac{R_{4,4-\text{days}}}{\text{Disturbance mortality rate}}=\frac{R_{4,4-\text{days}}}{0.01}$$

where *R*_*i*,*j*_ is the density of the resident on day *i* under *j*‐day disturbance treatment. Based on this calculation, we further assumed that *R*_8,16‐days_ = *R*_12,16‐days_ = *R*_4,16‐days_ for 16‐days disturbance treatment, where *R*_8,16‐days_ = *R* on day 8 in the 16‐day disturbance treatment and so forth. For 8‐days disturbance treatment, we assumed *R*_12,8‐days_ = *R*_4,8‐days_ and *R*_8,8‐days_ = 0.01 *R*_4,8‐days_ to account for the disturbance event on day 8.

In order to eliminate zero inflation, 1 was added to the final invader density *v* (post volume standardization) and was transformed to log(*v* + 1) to normalize the residuals. A value >0.69 (log[1 + 1]) would indicate that the invader increased in proportion throughout the experiment, whereas a value below this would suggest that invasion was unsuccessful.

To analyze the effect of disturbance and resource abundance on invasion success, *v*, a linear model was used to test effects of disturbance, resource abundance, and invader morphotype, with all two‐way and three‐way interactions. As the different morphotypes have distinct growth strategies, we expected their invasion success to be markedly different. Given a significant three‐way interaction in the most complex model, we did all further analysis on each invader morphotype (SM and WS) separately.

The biodiversity of the final resident populations (invader excluded) was calculated using the Simpson's index $D=1-\sum_{i}p_{i}^{2}$ where $p_{i}$ is the proportion of the *i*th morphotype (SM, WS, or FS) of the resident population (Simpson, 1949). This metric is commonly used to quantify diversity in this system (Buckling et al., 2000; Hall et al., 2012; Kassen et al., 2000).

For each invader morph, separate linear models were used to investigate treatment (disturbance frequency and resource abundance) effects on invasion success, evolved biodiversity and total resident density (log_10_[(cfu ml^−1^) + 1]). Previous work on this system has found a unimodal effect of disturbance on the diversity of *P. fluorescens* populations (Buckling et al., 2000). To understand how this effect of disturbance changes under different resource abundance, we treated disturbance frequency as a continuous predictor (with quadratic effect) and resource abundance as a categorical predictor. Treating resource abundance as a categorical predictor allowed us to easily interpret how the quadratic effect of disturbance changes under different resource abundances and allows comparisons to be made with previous work testing the effects of resources on diversity (Kassen et al., 2000). Model selection was done using likelihood ratio tests.

We then tested whether treatments indirectly affected invasion success through changes in resident populations. To do this, we first used a model with resident biodiversity and total resident density, plus their interaction, as predictors of invasion success. We then included treatment (disturbance, resource abundance, and their interaction), alongside resident population effects as predictors of success. The models with both treatment and resident population effects were initially tested using an ANOVA with type III sums of squares, then with type II if no significant interactions were found to account for differences in the ordering of predictors on significance testing.

Post‐hoc model comparisons were used to look at significant differences between levels of resource abundances and disturbance. For pairwise comparisons of single treatments (e.g., between high, medium, and low resource abundances), model estimates were averaged over other predictors in the model. Where multiple pairwise comparisons were used, *p* values were adjusted using Bonferroni adjustments. When comparing slopes to 0, confidence intervals overlapping zero indicated no significant effect. All statistical analyses were carried out in R version 4.0.3 (R Core Team, 2021).

## RESULTS

### Invasion success (invader proportional change) differed between invader types

**FIGURE 2:**
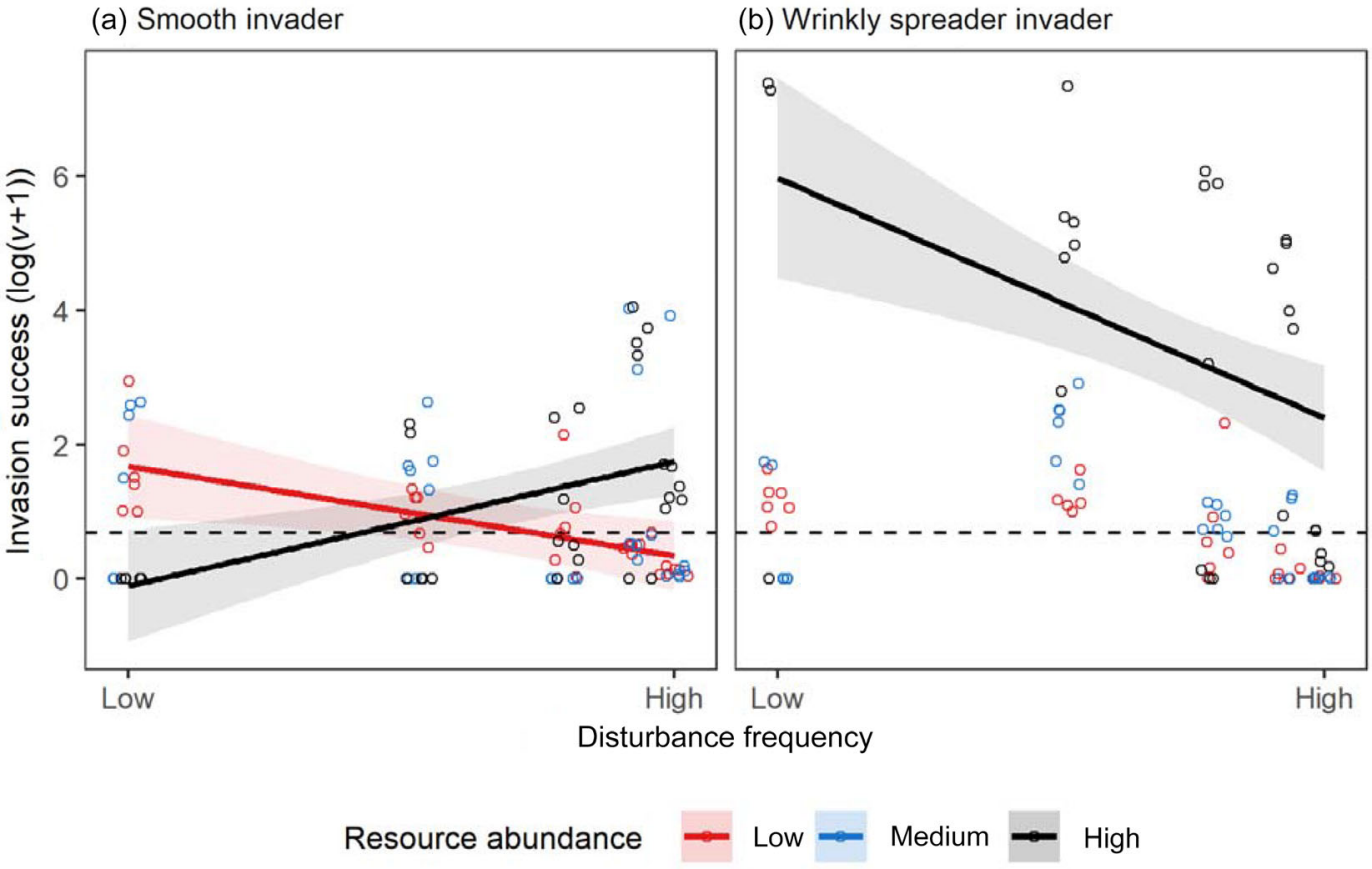
Invasion success, log(*v* + 1), of (a) the smooth (SM) invader and (b) the wrinkly spreader (WS), in response to different disturbance frequencies and resource abundances (low resources, red circles and lines; medium, blue; high, black). The variable *v* is the proportional change in invader density compared to the residents; the dashed line shows the value of equal population growth rate between residents and invaders, where invaders would have the same proportion in the community at the beginning and the end of the experiment. Jittered points represent individual replicates. Lines show the best model fits and shaded areas show the 95% confidence interval.

Invader success was significantly affected by a three‐way interaction between disturbance frequency, resource abundance, and invader morphotype (*F*_2,163_ = 10.2, *p* < 0.001; Figure 2). We therefore analyzed treatment effects on each invading morphotype separately (Appendix S1: Table S1). The fast‐growing smooth (SM) invaders were significantly affected by an interaction between disturbance frequency and resource abundance (*F*_2,85_ = 9.7, *p* < 0.001; Figure 2). Greater disturbance increased invasion success when resources were abundant (slope = 0.12, 95% CI [0.2, 0.050]), but decreased success when they were of low abundance (slope = −0.089, 95% CI [−0.020, −0.16]; Appendix S1: Table S2). Disturbance had no significant effect under medium resource abundance (slope = −0.048, 95% CI [0.021, −0.12]). This meant the highest levels of SM invasion occurred when both disturbance frequency and resource abundance was high.

Wrinkly spreader (WS) success was also affected by an interaction between disturbance frequency and resource abundance (*F*_2,78_ = 3.31, *p* = 0.042; Figure 2; Appendix S1: Table S1). Here we found disturbance to be negative for WS success when resources were high (slope = −0.24, 95% CI [−0.11, −0.37]), but to have no effect when they were at medium or low abundance (medium, slope = −0.033, 95% CI [0.069, −0.14]; low, slope = −0.076, 95% CI [0.026, −0.18]; Appendix S1: Table S2).

### Resident biodiversity was affected unimodally by disturbance, linearly by density

**FIGURE 3:**
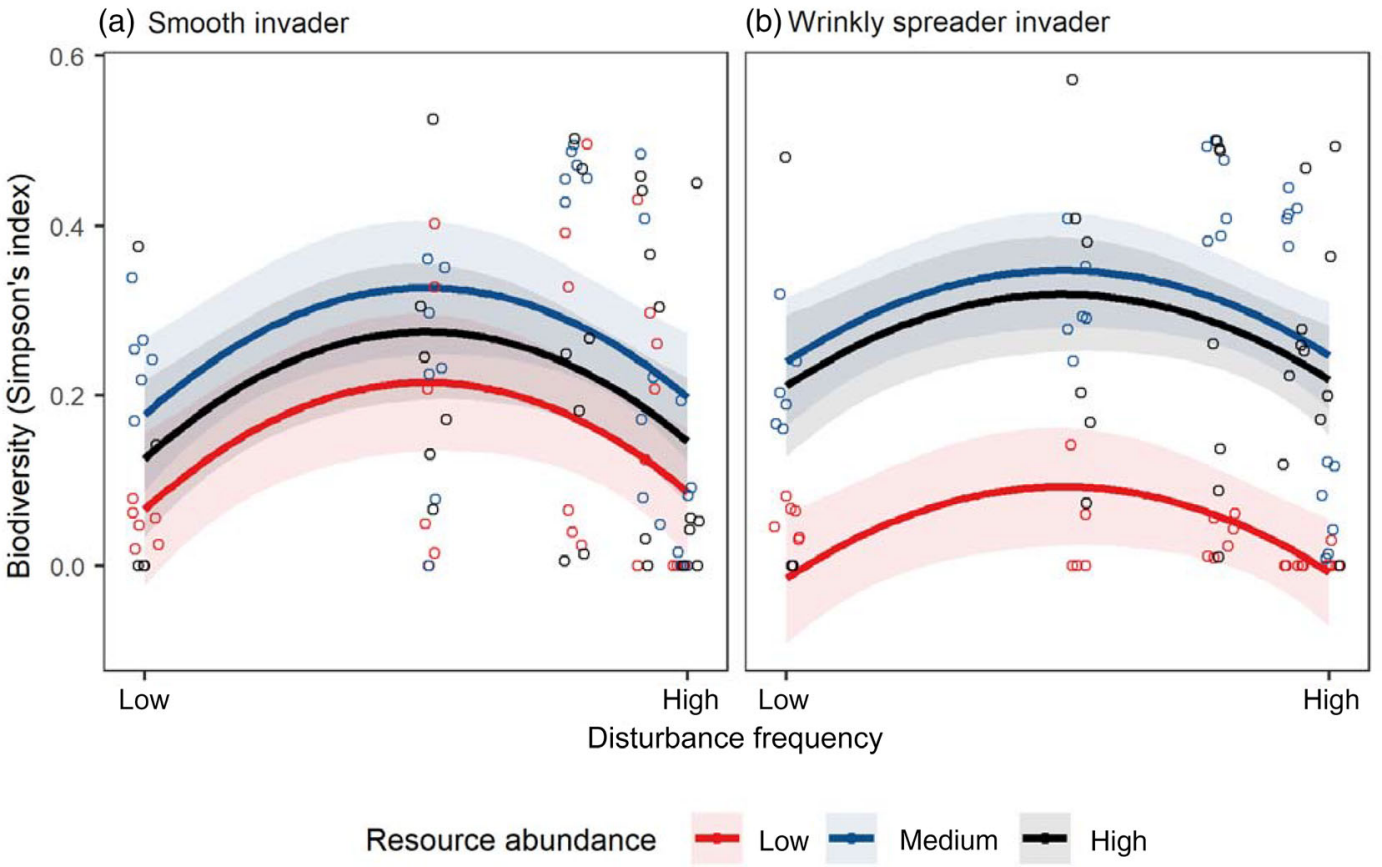
Evolved resident *Pseudomonas fluorescens* biodiversity (Simpson's index) in treatments of different disturbance frequencies (increasing from left to right within panels) and resource abundances (low resources, red circles and lines; medium, blue; high, black) when invaded by (a) a smooth (SM) invader and (b) a wrinkly spreader (WS). Diversity was significantly lower in the low resource treatment for both invaders. Resource abundance and invader type affected diversity through an interaction. Jittered points represent individual replicates. Lines show the best model fits and shaded areas show the 95% confidence interval.

Resident biodiversity (Simpson's index) showed the same unimodal pattern across disturbance frequencies irrespective of invader type (SM invader, *F*_1,86_ = 10.3, *p* = 0.002; WS, *F*_1,79_ = 7.87, *p* = 0.006) with the least diverse communities at both high and low disturbance (Figure 3). Resource abundance also altered resident biodiversity (SM invader, *F*_2,86_ = 3.84, *p* = 0.025; WS, *F*_2,79_ = 33.1, *p* < 0.001), with diversity being significantly lower in the low resource treatment than the medium when invaded by SM (*p* = 0.025) and lower than both the medium and high resource treatments when invaded by WS (*p* ≤ 0.001 for both).

**FIGURE 4:**
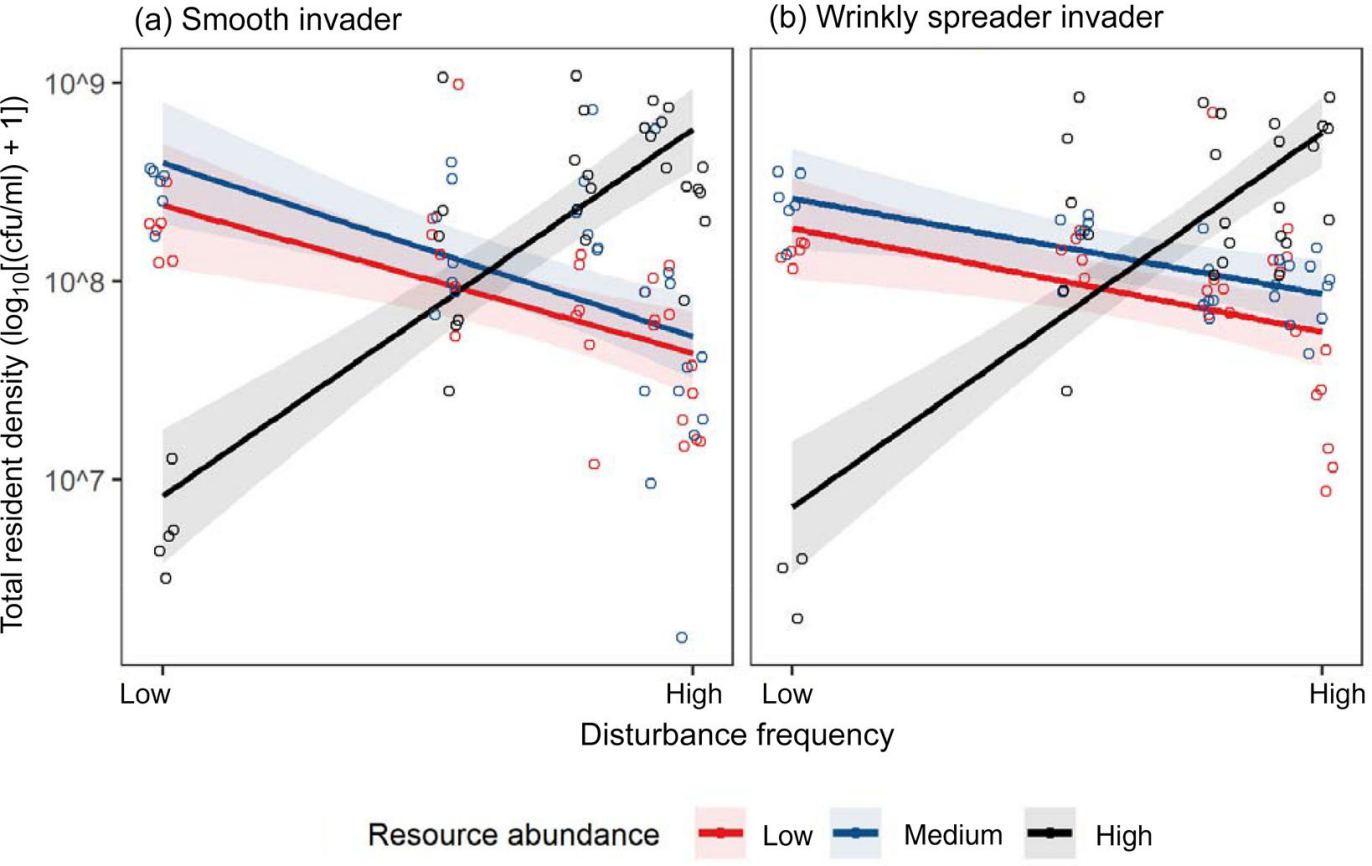
Final resident density (log_10_[(cfu/ml) + 1]) after 16 days in treatments of different resource abundances (low resources, red circles and lines; medium, blue; high, black and disturbance frequencies). Panel (a) shows treatments invaded with a smooth (SM) morphotype, panel (b) by a wrinkly spreader (WS). Jittered points represent individual replicates. Lines show the best model fits and shaded areas show the 95% confidence interval.

Like biodiversity, resident density showed the same patterns irrespective of invader type (Figure 4), with an interaction between disturbance frequency and resource abundance significantly affecting density (SM invader, *F*_2,85_ = 49.4, *p* < 0.001; WS, *F*_1,79_ = 47.0, *p* < 0.001; Figure 4). Resident density increased with disturbance under high resources, but disturbance negatively impacted density at low and medium resources (Figure 4).

### Resident population changes did not alter success above the direct effects of treatments

To test if these changes to the resident populations impacted invasion success, we first analyzed a model with resident biodiversity and total resident density, plus their interaction, as sole predictors of invasion success. Once again this was done separately for each invader morph. SM invaders were significantly affected by resident density (*F*_1,88_ = 5.03, *p* = 0.028), but not by biodiversity (*F*_1,88_ = 2.64, *p* = 0.11) or an interaction between density and diversity (*F*_1,87_ = 3.06, *p* = 0.084). Conversely, the WS invader was only significantly affected by biodiversity (*F*_1,81_ = 7.07, *p* = 0.010), with density having no significant effect either as a main effect (*F*_1,81_ = 0.67, *p* = 0.42) or through an interaction with biodiversity (*F*_1,80_ = 1.25, *p* = 0.27). This demonstrates treatments may have indirectly affected the success of both invaders by manipulating resident populations. We therefore tested whether the direct effect of treatments on success remained when these manipulations were considered. SM invaders were still significantly affected by the interaction between disturbance and resources (*F*_2,82_ = 9.27, *p* < 0.001). However, we find the effect of both biodiversity and total resident density to not be significant (biodiversity, *F*_1,82_ = 2.49, *p* = 0.12; density, *F*_1,82_ = 0.24, *p* = 0.63). When testing resident population effects alongside treatments on the success of the WS invader, we no longer found any significant interactions. Disturbance and resource abundance both significantly affected WS success (*F*_1,75_ = 8.27, *p* = 0.005 and *F*_2,75_ = 27.8, *p* < 0.001, respectively). However, resident population effects did not have a significant effect (biodiversity, *F*_1,75_ = 2.88, *p* = 0.094; total resident density, *F*_1,75_ = 0.006, *p* = 0.94). We therefore show that, although treatments had a significant effect on resident populations, this did not have an effect on success above the direct effects of disturbance and resource abundance.

## DISCUSSION

Here, we used a microbial system to experimentally test how disturbance frequency and resource abundance interact to affect the success of two ecologically different invaders. Both invaders were affected by an interaction between disturbance and resources, however this acted differently on each type of invader. The success of the fast‐growing smooth (SM) invader increased with increasing disturbance frequency when resources were abundant, but decreased when resources were low. Conversely, the slower growing wrinkly spreader (WS) suffered decreasing success with increasing disturbance frequency under high resource abundance but was not affected by disturbance in medium or low resource conditions.

Disturbances are commonly linked with invasion success (Altman & Whitlatch, 2007; Lear et al., 2020; Roxburgh et al., 2004; Shumway & Bertness, 1994), and the positive relationship between disturbance frequency and SM invasion success in the resource rich treatment supports this view. Disturbances open up resources for the fast‐growing invaders and reduce biotic resistance (Fargione et al., 2003; Fukami, 2015; Hodgson et al., 2002; Lear et al., 2020). Moreover, high resource availability allows rapid population growth between disturbances, reducing the chance of small invader populations being stochastically removed by disturbance. That SM invaders had reduced fitness at low disturbance frequency and high resource abundance was likely a consequence of escalating broth toxicity and oxygen depletion. Moreover, surviving residents may have reduced invader access to resources through priority and dominance effects (Hodgson et al., 2002; Zee & Fukami, 2018). These factors (broth toxicity, oxygen depletion, and resident effects) will likely be weaker when resources are less abundant as growth will be slower, potentially explaining why success was higher at low disturbance when resource abundance was lower. The inability of disturbances to facilitate invasion under lower resources can be explained by disturbances not providing sufficient additional resources to benefit the invader (Lear et al., 2020). At the lowest resource levels, the inhibitory effect of disturbance on invasion is presumably because invader populations could not grow fast enough between disturbances to recover. These results may offer an explanation as to why disturbance may not always facilitate invasion by fast‐growing colonizer species.

It is likely that low disturbance and high resource facilitated WS invasion because of its ecological niche: WS forms a mat at the air–broth interface that provides access to both nutrients and oxygen. Mat formation requires a threshold density to be reached, and low disturbance and high resource abundance will make this more likely (Brockhurst et al., 2006; Buckling et al., 2000; Hall et al., 2012). At higher disturbances and lower resources, the slower growth rate of WS relative to SM (Haddad et al., 2008) also likely increases the importance of stochastic removal of WS invaders, which would have happened less under high resources due to faster growth rates. We therefore demonstrate that high resource abundance can reduce the negative effects of disturbance on slower‐growing species. That the WS invader had much greater success than the faster growing SM under high resources and low disturbance shows the classical view that invaders are fast‐growing colonizer species (Mächler & Altermatt, 2012; van Kleunen et al., 2010) depends strongly on the new disturbance regime. This suggests the balance between disturbance‐induced mortality and growth rate is an important factor deciding invader success, with resource abundance dictating growth rate and disturbance affecting mortality.

As well as invader success, treatments affected resident populations, with disturbance and resources affecting resident biodiversity and total density. Consistent with previous work in this system, and with theory, we found a unimodal disturbance–diversity relationship (Benmayor et al., 2008; Buckling et al., 2000; Chesson, 2000; Chesson & Huntly, 1997). This relationship between disturbance and diversity was the same across resource treatments, but diversity was lower under the lowest resource abundance as also reported previously (Kassen et al., 2000, 2004; Hall & Colegrave, 2007). Resident density decreasing with increasing disturbance in low and medium (but not high) resource abundances is most is likely explained by resource‐limited growth causing slow population recovery between disturbances. Changes to resident populations were, however, found to have little indirect effect on invasion resistance, with their explanatory power nonsignificant when direct treatment effects were included in the model. This does not rule out a role for resident species but suggests that they were relatively unimportant compared with the direct effects of treatments. Further, we show factors that cause differences in biodiversity (for example disturbance frequency and resource abundance) need to be controlled for when studying the effect of diversity on invasion resistance, as the direct effect of these may cause the differences in success rather than biodiversity per se (as is the case of the SM invader here).

In conclusion, disturbance frequency and resource abundance both affected the success of two different invaders. Further, both invaders were differently affected by an interaction between these factors: the fast‐growing SM success was positively associated with disturbance frequency when resources were readily available, but negatively when they were limited, while the slower‐growing WS was only affected by disturbance when resource abundance was high. As this interaction between disturbance and resources was mediated through two fundamental processes, growth and mortality, we hypothesize that it may be broadly relevant. However, more empirical and theoretical studies are needed to understand how the processes underlying our system's response to disturbance could also drive the disturbance‐related patterns observed in more complex natural systems. Additionally, and contrary to conventional wisdom that invaders are generally fast‐growing species, the slower growing WS invader had very high success when disturbance was infrequent and resource abundance high. We therefore demonstrate that, when studying invasion ecology, multiple factors need to be considered to create an accurate predictive theory of invasibility, with the same disturbance frequency having both positive and negative effects depending on resource abundance and invader life history. Finally, we show that, by understanding these interactions, it may be possible through ecological manipulations of resource abundance to reduce the effect that disturbances have on invasion resistance.

## CONFLICT OF INTEREST

The authors declare no conflict of interest.

## Supporting information


Appendix S1

## Data Availability

Data (Lear et al., 2021a) are available in Zenodo at https://doi.org/10.5281/zenodo.5057319. R code used for all analysis and the creation of figures and tables (Lear et al., 2021b) is available in Zenodo at https://doi.org/10.5281/zenodo.6334795.

## References

[ecy3728-bib-0001] Agard, J. B. R. , R. H. Hubbard , and J. K. Griffith . 1996. “The Relation between Productivity, Disturbance and the Biodiversity of Caribbean Phytoplankton: Applicability of Huston's Dynamic Equilibrium Model.” Journal of Experimental Marine Biology and Ecology 202(1): 1–17.

[ecy3728-bib-0002] Altman, S. , and R. B. Whitlatch . 2007. “Effects of Small‐Scale Disturbance on Invasion Success in Marine Communities.” Journal of Experimental Marine Biology and Ecology 342(1): 15–29.

[ecy3728-bib-0003] Baldwin, D. S. , and A. M. Mitchell . 2000. “The Effects of Drying and Re‐flooding on the Sediment and Soil Nutrient Dynamics of Lowland River–Floodplain Systems: A Synthesis.” Regulated Rivers: Research & Management 16(5): 457–67.

[ecy3728-bib-0004] Benmayor, R. , A. Buckling , M. B. Bonsall , M. A. Brockhurst , and D. J. Hodgson . 2008. “The Interactive Effects of Parasites, Disturbance, and Productivity on Experimental Adaptive Radiations.” Evolution: International Journal of Organic Evolution 62(2): 467–77. 18039322 10.1111/j.1558-5646.2007.00268.x

[ecy3728-bib-0005] Brockhurst, M. A. , M. E. Hochberg , T. Bell , and A. Buckling . 2006. “Character Displacement Promotes Cooperation in Bacterial Biofilms.” Current Biology 16(20): 2030–4. 17055982 10.1016/j.cub.2006.08.068

[ecy3728-bib-0006] Buckling, A. , R. Kassen , G. Bell , and P. B. Rainey . 2000. “Disturbance and Diversity in Experimental Microcosms.” Nature 408(6815): 961. 11140680 10.1038/35050080

[ecy3728-bib-0007] Chesson, P. 2000. “Mechanisms of Maintenance of Species Diversity.” Annual Review of Ecology and Systematics 31(1): 343–66.

[ecy3728-bib-0008] Chesson, P. , and N. Huntly . 1997. “The Roles of Harsh and Fluctuating Conditions in the Dynamics of Ecological Communities.” The American Naturalist 150(5): 519–53. 10.1086/286080 18811299

[ecy3728-bib-0009] Davis, M. A. , J. Philip Grime , and K. Thompson . 2000. “Fluctuating Resources in Plant Communities: A General Theory of Invasibility.” Journal of Ecology 88(3): 528–34.

[ecy3728-bib-0010] Didham, R. K. , J. M. Tylianakis , M. A. Hutchison , R. M. Ewers , and N. J. Gemmell . 2005. “Are Invasive Species the Drivers of Ecological Change?” Trends in Ecology & Evolution 20(9): 470–4. 16701420 10.1016/j.tree.2005.07.006

[ecy3728-bib-0011] Emery, S. M. , and K. L. Gross . 2007. “Dominant Species Identity, Not Community Evenness, Regulates Invasion in Experimental Grassland Plant Communities.” Ecology 88(4): 954–64. 17536711 10.1890/06-0568

[ecy3728-bib-0012] Fargione, J. , C. S. Brown , and D. Tilman . 2003. “Community Assembly and Invasion: An Experimental Test of Neutral Versus Niche Processes.” Proceedings of the National Academy of Sciences USA 100(15): 8916–20. 10.1073/pnas.1033107100 PMC166413 12843401

[ecy3728-bib-0013] Fausch, K. D. , Y. Taniguchi , S. Nakano , G. D. Grossman , and C. R. Townsend . 2001. “Flood Disturbance Regimes Influence Rainbow Trout Invasion Success among Five Holarctic Regions.” Ecological Applications 11(5): 1438–55.

[ecy3728-bib-0014] Fridley, J. D. , J. J. Stachowicz , S. Naeem , D. F. Sax , E. W. Seabloom , M. D. Smith , T. J. Stohlgren , D. Tilman , and B. Von Holle . 2007. “The Invasion Paradox: Reconciling Pattern and Process in Species Invasions.” Ecology 88(1): 3–17. 17489447 10.1890/0012-9658(2007)88[3:tiprpa]2.0.co;2

[ecy3728-bib-0015] Fukami, T. 2015. “Historical Contingency in Community Assembly: Integrating Niches, Species Pools, and Priority Effects.” Annual Review of Ecology, Evolution, and Systematics 46: 1–23.

[ecy3728-bib-0016] Galand, P. E. , S. Lucas , S. K. Fagervold , E. Peru , A. M. Pruski , G. Vétion , C. Dupuy , and K. Guizien . 2016. “Disturbance Increases Microbial Community Diversity and Production in Marine Sediments.” Frontiers in Microbiology 7: 1950. 27994581 10.3389/fmicb.2016.01950 PMC5133735

[ecy3728-bib-0017] Gómez, P. , and A. Buckling . 2013. “Real‐Time Microbial Adaptive Diversification in Soil.” Ecology Letters 16(5): 650–5. 23438288 10.1111/ele.12093

[ecy3728-bib-0018] Green, P. T. , D. J. O'Dowd , K. L. Abbott , M. Jeffery , K. Retallick , and R. M. Nally . 2011. “Invasional Meltdown: Invader–Invader Mutualism Facilitates a Secondary Invasion.” Ecology 92(9): 1758–68. 21939072 10.1890/11-0050.1

[ecy3728-bib-0019] Haddad, N. M. , M. Holyoak , T. M. Mata , K. F. Davies , B. A. Melbourne , and K. Preston . 2008. “Species’ Traits Predict the Effects of Disturbance and Productivity on Diversity.” Ecology Letters 11(4): 348–56. 18201199 10.1111/j.1461-0248.2007.01149.x

[ecy3728-bib-0020] Hall, A. R. , and N. Colegrave . 2007. “How Does Resource Supply Affect Evolutionary Diversification?” Proceedings of the Royal Society B: Biological Sciences 274(1606): 73–8. 10.1098/rspb.2006.3703 PMC1679875 17015335

[ecy3728-bib-0021] Hall, A. R. , A. D. Miller , H. C. Leggett , S. H. Roxburgh , A. Buckling , and K. Shea . 2012. “Diversity–Disturbance Relationships: Frequency and Intensity Interact.” Biology Letters 8(5): 768–71. 22628097 10.1098/rsbl.2012.0282 PMC3440969

[ecy3728-bib-0022] Hobbs, R. J. , and L. F. Huenneke . 1992. “Disturbance, Diversity, and Invasion: Implications for Conservation.” Conservation Biology 6(3): 324–37.

[ecy3728-bib-0023] Hodgson, D. J. , P. B. Rainey , and A. Buckling . 2002. “Mechanisms Linking Diversity, Productivity and Invasibility in Experimental Bacterial Communities.” Proceedings of the Royal Society B 269(1506): 2277–83. 12427320 10.1098/rspb.2002.2146 PMC1691149

[ecy3728-bib-0024] Kassen, R. , A. Buckling , G. Bell , and P. B. Rainey . 2000. “Diversity Peaks at Intermediate Productivity in a Laboratory Microcosm.” Nature 406(6795): 508. 10952310 10.1038/35020060

[ecy3728-bib-0025] Kassen, R. , M. Llewellyn , and P. B. Rainey . 2004. “Ecological Constraints on Diversification in a Model Adaptive Radiation.” Nature 431(7011): 984. 15496923 10.1038/nature02923

[ecy3728-bib-0026] Koza, A. , O. Moshynets , W. Otten , and A. J. Spiers . 2011. “Environmental Modification and Niche Construction: Developing O_2_ Gradients Drive the Evolution of the Wrinkly Spreader.” The ISME Journal 5(4): 665. 20962880 10.1038/ismej.2010.156 PMC3105741

[ecy3728-bib-0027] Lake, J. C. , and M. R. Leishman . 2004. “Invasion Success of Exotic Plants in Natural Ecosystems: The Role of Disturbance, Plant Attributes and Freedom from Herbivores.” Biological Conservation 117(2): 215–26.

[ecy3728-bib-0028] Lake, P. S. 2000. “Disturbance, Patchiness, and Diversity in Streams.” Journal of the North American Benthological Society 19(4): 573–92.

[ecy3728-bib-0029] Lear, L. , D. Padfield , H. Inamine , K. Shea , and A. J. Buckling . 2021a. “Disturbance‐Mediated Invasions are Dependent on Community Resource Abundance [Data Set].” Zenodo. 10.5281/zenodo.5057319. PMC9542494 35412647

[ecy3728-bib-0030] Lear, L. , D. Padfield , H. Inamine , K. Shea , and A. J. Buckling . 2021b. “Disturbance‐Mediated Invasions are Dependent on Community Resource Abundance: Code.” Zenodo. 10.5281/zenodo.6334795. PMC9542494 35412647

[ecy3728-bib-0031] Lear, L. , E. Hesse , K. Shea , and A. Buckling . 2020. “Disentangling the Mechanisms Underpinning Disturbance‐Mediated Invasion.” Proceedings of the Royal Society B 287(1919): 20192415. 31992171 10.1098/rspb.2019.2415 PMC7015320

[ecy3728-bib-0032] Leishman, M. R. , T. Haslehurst , A. Ares , and Z. Baruch . 2007. “Leaf Trait Relationships of Native and Invasive Plants: Community‐and Global‐Scale Comparisons.” New Phytologist 176(3): 635–43. 17822409 10.1111/j.1469-8137.2007.02189.x

[ecy3728-bib-0033] Lembrechts, J. J. , A. Pauchard , J. Lenoir , M. A. Nuñez , C. Geron , A. Ven , P. Bravo‐Monasterio , E. Teneb , I. Nijs , and A. Milbau . 2016. “Disturbance Is the Key to Plant Invasions in Cold Environments.” Proceedings of the National Academy of Sciences USA 113(49): 14061–6. 10.1073/pnas.1608980113 PMC5150417 27872292

[ecy3728-bib-0034] Levine, J. M. , and C. M. D'Antonio . 1999. “Elton Revisited: A Review of Evidence Linking Diversity and Invasibility.” Oikos 87: 15–26.

[ecy3728-bib-0035] Levine, J. M. , M. Vilà , C. M. D'Antonio , J. S. Dukes , K. Grigulis , and S. Lavorel . 2003. “Mechanisms Underlying the Impacts of Exotic Plant Invasions.” Proceedings of the Royal Society B 270(1517): 775–81. 10.1098/rspb.2003.2327. 12737654 PMC1691311

[ecy3728-bib-0036] Mächler, E. , and F. Altermatt . 2012. “Interaction of Species Traits and Environmental Disturbance Predicts Invasion Success of Aquatic Microorganisms.” PLoS One 7(9): e45400. 23028985 10.1371/journal.pone.0045400 PMC3447880

[ecy3728-bib-0037] Miller, A. D. , H. Inamine , A. Buckling , S. H. Roxburgh , and K. Shea . 2021. “How Disturbance History Alters Invasion Success: Biotic Legacies and Regime Change.” Ecology Letters 24(4): 687–97. 33506576 10.1111/ele.13685 PMC8048489

[ecy3728-bib-0038] Naeem, S. , J. M. H. Knops , D. Tilman , K. M. Howe , T. Kennedy , and S. Gale . 2000. “Plant Diversity Increases Resistance to Invasion in the Absence of Covarying Extrinsic Factors.” Oikos 91(1): 97–108.

[ecy3728-bib-0039] Narimanov, N. , A. Kempel , M. van Kleunen , and M. H. Entling . 2020. “Unexpected Sensitivity of the Highly Invasive Spider Mermessus Trilobatus to Soil Disturbance in Grasslands.” Biological Invasions 23: 1–6. 33488272 10.1007/s10530-020-02348-9 PMC7801346

[ecy3728-bib-0040] O'Dowd, D. J. , P. T. Green , and P. S. Lake . 2003. “Invasional ‘meltdown' on an Oceanic Island.” Ecology Letters 6(9): 812–7.

[ecy3728-bib-0041] R Core Team . 2021. R: A Language and Environment for Statistical Computing. Vienna: R Foundation for Statistical Computing. www.R-project.org.

[ecy3728-bib-0042] Rainey, P. B. , and M. Travisano . 1998. “Adaptive Radiation in a Heterogeneous Environment.” Nature 394(6688): 69. 9665128 10.1038/27900

[ecy3728-bib-0043] Ricciardi, A. 2001. “Facilitative Interactions among Aquatic Invaders: Is an "Invasional Meltdown" Occurring in the Great Lakes?” Canadian Journal of Fisheries and Aquatic Sciences 58(12): 2513–25.

[ecy3728-bib-0044] Ross‐Gillespie, A. , A. Gardner , S. A. West , and A. S. Griffin . 2007. “Frequency Dependence and Cooperation: Theory and a Test with Bacteria.” The American Naturalist 170(3): 331–42. 10.1086/519860 17879185

[ecy3728-bib-0045] Roxburgh, S. H. , K. Shea , and J. Bastow Wilson . 2004. “The Intermediate Disturbance Hypothesis: Patch Dynamics and Mechanisms of Species Coexistence.” Ecology 85(2): 359–71.

[ecy3728-bib-0046] Seabloom, E. W. , W. Stanley Harpole , O. J. Reichman , and D. Tilman . 2003. “Invasion, Competitive Dominance, and Resource Use by Exotic and Native California Grassland Species.” Proceedings of the National Academy of Sciences USA 100(23): 13384–9. 10.1073/pnas.1835728100 PMC263823 14595028

[ecy3728-bib-0047] Shea, K. , and P. Chesson . 2002. “Community Ecology Theory as a Framework for Biological Invasions.” Trends in Ecology & Evolution 17(4): 170–6.

[ecy3728-bib-0048] Sher, A. A. , and L. A. Hyatt . 1999. “The Disturbed Resource‐Flux Invasion Matrix: A New Framework for Patterns of Plant Invasion.” Biological Invasions 1(2–3): 107–14.

[ecy3728-bib-0049] Shumway, S. W. , and M. D. Bertness . 1994. “Patch Size Effects on Marsh Plant Secondary Succession Mechanisms.” Ecology 75(2): 564–8.

[ecy3728-bib-0050] Simberloff, D. , and B. Von Holle . 1999. “Positive Interactions of Nonindigenous Species: Invasional Meltdown?” Biological Invasions 1(1): 21–32.

[ecy3728-bib-0051] Simpson, E. H. 1949. “Measurement of Diversity.” Nature 163(4148): 688–8.

[ecy3728-bib-0052] Spiers, A. J. , S. G. Kahn , J. Bohannon , M. Travisano , and P. B. Rainey . 2002. “Adaptive Divergence in Experimental Populations of *Pseudomonas fluorescens*. I. Genetic and Phenotypic Bases of Wrinkly Spreader Fitness.” Genetics 161(1): 33–46. 12019221 10.1093/genetics/161.1.33 PMC1462107

[ecy3728-bib-0053] Stachowicz, J. J. , H. Fried , R. W. Osman , and R. B. Whitlatch . 2002. “Biodiversity, Invasion Resistance, and Marine Ecosystem Function: Reconciling Pattern and Process.” Ecology 83(9): 2575–90.

[ecy3728-bib-0054] Tilman, D. 2004. “Niche Tradeoffs, Neutrality, and Community Structure: A Stochastic Theory of Resource Competition, Invasion, and Community Assembly.” Proceedings of the National Academy of Sciences USA 101(30): 10854–61. 10.1073/pnas.0403458101 PMC503710 15243158

[ecy3728-bib-0055] van Kleunen, M. , E. Weber , and M. Fischer . 2010. “A Meta‐Analysis of Trait Differences between Invasive and Non‐invasive Plant Species.” Ecology Letters 13(2): 235–45. 20002494 10.1111/j.1461-0248.2009.01418.x

[ecy3728-bib-0056] Vitousek, P. M. , C. M. D'Antonio , L. L. Loope , M. Rejmanek , and R. Westbrooks . 1997. “Introduced Species: A Significant Component of Human‐Caused Global Change.” New Zealand Journal of Ecology 21(1): 1–16.

[ecy3728-bib-0057] Worm, B. , H. K. Lotze , H. Hillebrand , and U. Sommer . 2002. “Consumer Versus Resource Control of Species Diversity and Ecosystem Functioning.” Nature 417(6891): 848–51. 12075351 10.1038/nature00830

[ecy3728-bib-0058] Zee, P. C. , and T. Fukami . 2018. “Priority Effects Are Weakened by a Short, but Not Long, History of Sympatric Evolution.” Proceedings of the Royal Society B 285(1871): 20171722. 29386363 10.1098/rspb.2017.1722 PMC5805921

[ecy3728-bib-0059] Zhang, Q.‐G. , and A. Buckling . 2016. “Migration Highways and Migration Barriers Created by Host–Parasite Interactions.” Ecology Letters 19(12): 1479–85. 27873470 10.1111/ele.12700

[ecy3728-bib-0060] Zhang, X. , E. R. Johnston , A. Barberán , Y. Ren , Z. Wang , and X. Han . 2018. “Effect of Intermediate Disturbance on Soil Microbial Functional Diversity Depends on the Amount of Effective Resources.” Environmental Microbiology 20(10): 3862–75. 30209865 10.1111/1462-2920.14407

[ecy3728-bib-0061] Zhang, X.‐X. , and P. B. Rainey . 2007. “Construction and Validation of a Neutrally‐Marked Strain of *Pseudomonas fluorescens* SBW25.” Journal of Microbiological Methods 71(1): 78–81. 17669526 10.1016/j.mimet.2007.07.001

