# Supplementary material for: Disturbance‐mediated invasions are dependent on community resource abundance
Source: Ecology. 2022 Jun 1;103(8):e3728. doi: 10.1002/ecy.3728 (PMC9542494; doi:10.1002/ecy.3728)
Supplement: Supplementary file 1 — Appendix S1 [file ECY-103-e3728-s001.pdf]

Supporting Information. Disturbance-mediated invasions are dependent on community resource abundance. Luke Lear, Daniel Padfield, Hidetoshi Inamine, Katriona Shea, and Angus Buckling. *Ecology*.

Appendix S1

Table S1 Coefficients from models testing the interactive effects of disturbance frequency (a continuous variable with five levels) and resource abundances (three levels: low, medium or high) on the success of two different invading morphotypes (Smooth and Wrinkly spreader, each analysed separately). Negative disturbance trends show increasing disturbance frequency reduced invader success, positive disturbance trends that the invader benefited. Trends are regarded as statistically significant if the upper and lower CL values do not cross 1.

Resource abundance	Disturbance trend	Degrees of freedom	Lower CL	Upper CL
Smooth invader				
low	0.0889	85	0.0201	0.1578
medium	0.0482	85	-0.0206	0.1169
high	-0.1236	85	-0.1964	-0.0507
Wrinkly spreader invader				
low	0.0761	78	-0.0257	0.1780
medium	0.0332	78	-0.0685	0.1350
high	0.2379	78	0.1104	0.3650

Table S2 Contrasts in the effect of disturbance frequency between resource abundances on the success of two invading morphotypes (Smooth and Wrinkly spreader). * signifies p=<0.05, ** p=<0.01 and ** p=<0.001.

Contrasts between resources	Estimate	Degrees of freedom	P value
Smooth invader			
low-medium	-0.041	85	0.684
low-high	-0.212	85	<0.001***
medium-high	-0.172	85	0.003**
Wrinkly spreader invader			
low-medium	-0.043	78	0.824
low-high	0.162	78	0.126
medium-high	0.205	78	0.038*
